# Structure and function analysis of a potent human neutralizing antibody CA521^FALA^ against SARS-CoV-2

**DOI:** 10.1038/s42003-021-02029-w

**Published:** 2021-04-23

**Authors:** Deyong Song, Wenbo Wang, Chuangchuang Dong, Zhenfei Ning, Xiu Liu, Chuan Liu, Guangying Du, Chunjie Sha, Kailin Wang, Jun Lu, Baiping Sun, Yanyan Zhao, Qiaoping Wang, Hongguang Xu, Ying Li, Zhenduo Shen, Jie Jiao, Ruiying Wang, Jingwei Tian, Wanhui Liu, Lan Wang, Yong-Qiang Deng, Changlin Dou

**Affiliations:** 1 Antibody Research and Development Center, Shandong Boan Biotechnology Co., Ltd., Yantai, China; 2 Division of Monoclonal Antibodies, Institute for Biological Product Control, National Institutes for Food and Drug Control (NIFDC), Beijing, China; 3 Shuimu BioSciences Ltd., Beijing, China; 4 State Key Laboratory of Long-acting and Targeting Drug Delivery System, Shandong Luye Pharmaceutical Co. Ltd., Yantai, China; 5 State Key Laboratory of Pathogen and Biosecurity, Institute of Microbiology and Epidemiology, Academy of Military Medical Sciences, Beijing, China

**Keywords:** Microbiology, Immunology

## Abstract

Severe acute respiratory syndrome coronavirus 2 (SARS-CoV-2) is the causative agent of the ongoing COVID-19 pandemic, which has resulted in more than two million deaths at 2021 February . There is currently no approved therapeutics for treating COVID-19. The SARS-CoV-2 Spike protein is considered a key therapeutic target by many researchers. Here we describe the identification of several monoclonal antibodies that target SARS-CoV-2 Spike protein. One human antibody, CA521^FALA^, demonstrated neutralization potential by immunizing human antibody transgenic mice. CA521^FALA^ showed potent SARS-CoV-2-specific neutralization activity against SARS-CoV-2 pseudovirus and authentic SARS-CoV-2 infection in vitro. CA521^FALA^ also demonstrated having a long half-life of 9.5 days in mice and 9.3 days in rhesus monkeys. CA521^FALA^ inhibited SARS-CoV-2 infection in SARS-CoV-2 susceptible mice at a therapeutic setting with virus titer of the lung reduced by 4.5 logs. Structural analysis by cryo-EM revealed that CA521^FALA^ recognizes an epitope overlapping with angiotensin converting enzyme 2 (ACE2)-binding sites in SARS-CoV-2 RBD in the Spike protein. CA521^FALA^ blocks the interaction by binding all three RBDs of one SARS-CoV-2 spike trimer simultaneously. These results demonstrate the importance for antibody-based therapeutic interventions against COVID-19 and identifies CA521^FALA^ a promising antibody that reacts with SARS-CoV-2 Spike protein to strongly neutralize its activity.

## Abstract (web summary)

Song et al describe and identify monoclonal antibodies that target the SARSCoV-2 Spike protein, including CA521FALA, which demonstrated neutralising potential in vivo and in vitro. They performed structural analysis, which revealed that CA521FALA recognizes an epitope overlapping with angiotensin converting enzyme 2 (ACE2)-binding sites in SARS-CoV-2 Spike protein therefore making it a promising therapeutic antibody.

## Introduction

World Health Organization as of February 18th in 2021 has reported that the coronavirus disease 2019 (COVID-19) caused by the virus severe acute respiratory syndrome coronavirus 2 (SARS-CoV-2) has caused 2,424,060 deaths globally (https://www.who.int/). SARS-CoV-2 is closely related to SARS-CoV and belongs to the lineage B of the genus *Betacoronavirus* in the *Coronaviridae* family. There are no therapeutics approved for the treatment of COVID-19. Monoclonal antibodies (MAbs) are promising candidates to combat emerging viruses. For example, in the case of the Ebola virus, MAbs MAb114 and REGN-EB3 have shown striking treatment benefits, reducing the mortality rate from ~67% to 33.5–35.1% for all patients and to 9.9–11.2% for patients with low viral loads^1^. MAbs are also being considered as promising therapeutics for COVID-19 patients^2^.

Sharing an amino acid sequence identity of ~80% in the envelope-located spike glycoprotein (Sike protein), both SARS-CoV-2 and SARS-CoV use human angiotensin-converting enzyme 2 (hACE2) to enter host cells. Cellular entry is achieved by the homotrimeric S-mediated virus-receptor engagement through the receptor-binding domain (RBD) followed by virus-host membrane fusion^3–7^. The Spike protein is a viral factor that mediates attachment to cells and fusion of the viral and cellular membrane, functions associated with the S1 and S2 subunits, respectively^3–7^. The primary amino acid sequence of the S1 subunit of coronaviruses determines host receptors. The structure and function of the SARS CoV-2 Spike protein have been determined^8^. S protein trimer of coronavirus S structures always has some RBDs in an “up” conformation and some in the “down” conformation^9–12^. 3D structures of SARS-CoV-2 S protein also reveal some RBDs in “up” conformations and some in the “down” conformation^8,13–15^. The crystal structure of the RBD of the spike protein of SARS-CoV-2 bound to the cell receptor ACE2 has also been determined. This structural analysis identified the residues of the RBD that are essential for ACE2 binding^16^.

Potent neutralizing antibodies including S230, m396, 80R, CR3022 have been shown to target the SARS-CoV RBD in S1, disable receptor interactions. Unfortunately, none demonstrates potent neutralizing activity against SARS-CoV-2^12,14,17–20^. In addition, previous studies have shown that pre-existing serum antibodies associate with poor outcomes in patients with the 2009 influenza infection or SARS-CoV infection^21–23^. This may be due in part to antibody-dependent enhancement (ADE) which could lead to acute respiratory injury and is a potential risk for antibody therapeutic developed against SARS-CoV infection^24^. ADE has been shown in part to be induced mainly through engaging viruses and monocytes by anti-virus antibody binding with Fc Receptor or complement receptors on monocytes^24–30^. Recent studies show that sera from the COVID-19 patients with the severe disease have higher NAb titer than sera from mild or asymptomatic patient^31^. Given the highly phylogenetic relationship between SARS-CoV and SARS-CoV-2, the risk of ADE will likely need to be mitigated for anti-SARS-CoV-2 development. IgG4 backbone is often used to attenuate interactions with the Fc receptors or complement receptors. Several modifications to the Fc amino acid sequence can be used to further eliminate their interaction with FcγRI and FcγRII. The L234A/L235A mutation (L234A/L235A for IgG1 backbone, but F234A/L235A for IgG4 backbone) is among the most commonly used modifications^32–37^.

Here, we report the isolation of several highly potent neutralizing MAbs against SARS-CoV-2 from human antibody transgenic mice. Given that ADE is a potential risk for antibody therapeutics against SARS-CoV-2 infection we introduced FALA mutation to potentially abrogate the effect in these neutralizing MAbs. One human MAb designated CA521^FALA^ demonstrated potent SARS-CoV-2-specific neutralization activity in vitro and in vivo and had no risk of ADE. Pharmacokinetics revealed that CA521^FALA^ is also stable in serum of mice and rhesus monkeys. Cryo-electron microscopy characterization of the complexes formed by Spike protein and CA521^FALA^ IgG/Fab showed that it recognizes a patch of residues overlapping with angiotensin-converting enzyme 2 (ACE2)-binding sites in the SARS-CoV-2 receptor-binding domain (RBD) of the Spike protein. Structural analysis reveals that the CA521^FALA^ can block the interaction by binding all three RBDs of one SARS-CoV-2 spike protein simultaneously whether it was in “up” or “down” conformations and the cryo-EM structures provided evidence of bivalent binding of full-length IgG with two adjacent RBDs.

## Results

### MAb CA521^FALA^ can block binding of SARS-CoV-2-RBD to hACE2 receptor and specifically bind the Spike protein of SARS-CoV-2

Potential SARS-CoV-2 targeting antibodies were identified by screening immunized human antibody transgenic mice by phage display (Supplementary Fig. 1). For these three types of mouse, immunization was used: (1) two mice were immunized with recombinant Spike ectodomain protein (S1 + S2); (2) two mice were immunized with a mixture of Spike S1 protein and RBD protein; (3) three mice were immunized with the mixture of recombinant RBD protein, Spike S1 protein and Spike S2 protein (For details, please see Materials and Methods and Supplementary Table 1). Serum antibody titer was detected by enzyme-linked immunosorbent assay (ELISA) after three rounds of immunization (Supplementary Fig. 2). These antigens were also used as the baits for screening the phage antibody library. Sixty clones that showed blocking activity as ScFvs with different sequences were expressed as full IgGs (IgG4 backbone, F234A/L235A mutation was introduced into various targets to abrogated or eliminate the binding with Fc receptors or complement receptors). Interestingly, all 60 clones were derived from mice immunized with a mixture of recombinant proteins (immunization type 2 and 3), but not ectodomain protein (immunization type 1). These results may indicate this Spike ectodomain protein from insect cells is not a good immunogen for getting clones with receptor blocking activity (Supplementary Table 1).

After blocking activity analysis by ELISA-based receptor-binding inhibition assay, 34 candidates were selected for pseudovirus neutralization evaluation with 293T-ACE2 cells as target cells and 7 candidates showed lower IC50. Further evaluation for the seven candidates was performed, such as antibody yield in 293F cells, pseudovirus neutralization evaluation with Huh-7 cells as target cells, and pharmacokinetics in the mouse. CA521^FALA^ was chosen as the lead candidate as it outperformed the other candidates (Supplementary Fig. 3).

**Fig. 1:**
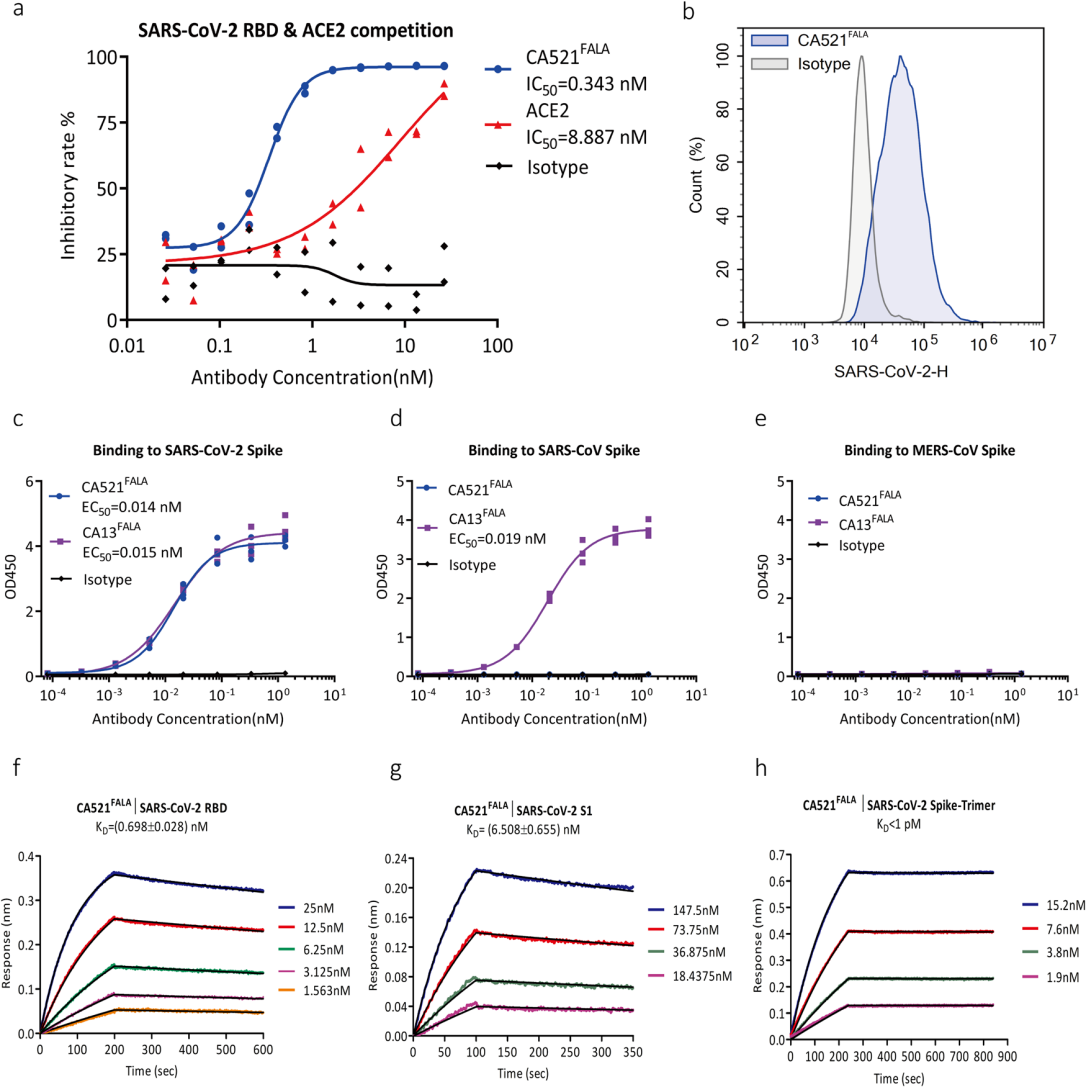
CA521^FALA^ can block the binding of SARS-CoV-2-RBD to hACE2 receptor and specifically bind Spike of SARS-CoV-2. **a** CA521^FALA^ can effectively block RBD binding to ACE2 receptor in ELISA. CA521^FALA^ and hACE2 protein can block the binding of SARS-CoV-2 RBD and hACE2 with IC50 of 0.343 and 8.887 nM, respectively. Experiments were performed in duplicate, value = mean ± SD. **b** CA521^FALA^ could specifically bind to CHO-K1 cells expressing SARS-CoV-2 Spike. SARS-CoV-2 Spike protein transfected CHO-K1 cells were stained with isotype control, CA521^FALA^ at a concentration of 0.74 μg/mL. FITC-anti-HuFc secondary antibody was used for flow cytometry. Irrelevant mAb with the same constant region of CA521^FALA^ was used as an isotype. Experiments were performed in triplicate and one representative data was displayed. **c**–**e** CA521^FALA^ could specifically bind to SARS-CoV-2 Spike protein, but does not cross-react with SARS-CoV Spike or MERS-CoV Spike protein in Elisa. CA521^FALA^ binds SARS-CoV-2 Spike protein with EC50 of 0.014 nM. CA13, which is an anti- SARS-CoV-2 S2 domain mAb, can bind Spike of SARS-CoV-2 and SARS-CoV with EC50 of 0.015 and 0.019 nM. Experiments were performed in triplicate, value = Mean ± SD. **f**–**h** The binding kinetics of CA521^FALA^ were assessed by biolayer Interferometry (BLI) assay using the Octet RED96 system (FortéBio). Trimer protein is from Shuimu BioSciences. Experiments were performed three times and one representative data was displayed.

Further analysis demonstrated that CA521 ^FALA^ blocked the binding of SARS-CoV-2-RBD with recombinant hACE2 receptor strongly compared to hACE2 protein, with IC50 as 0.343 and 8.887 nM, respectively (Fig. 1a). CA521^FALA^ also showed the ability to block the binding of recombinant ACE2 to SARS-CoV-2 Spike expressing 293F cells and the binding of SARS-CoV-2 RBD to ACE2 expressing 293T cells (Supplementary Fig. 4). Flow cytometry (FACS) experiments revealed that CA521^FALA^ could also specifically bind to SARS-CoV-2 Spike protein transfected CHO-K1 cells (Fig. 1b). Gating strategy for flow cytometry was shown in Supplementary Fig. 5. CA521^FALA^ could bind SARS-CoV-2-Spike specifically and not cross-react with SARS-CoV and MERS-CoV Spike (Fig. 1c–e). Biolayer interferometry (BLI) using the Octet RED96 system (FortéBio) assessed the binding kinetics of CA521^FALA^. The measured equilibrium constant (KD) of CA521^FALA^ with SARS-CoV-2-RBD, S1, and spike trimer (Shuimu BioSciences Ltd.) was 0.698 ± 0.028 nM, 6.508 ± 0.655 nM, and <1 pM (below detection limit), respectively (Fig. 1f–h).

### CA521 ^FALA^ shows modified binding affinity to various Fc or complement receptors in BIAcore or cell-based assay

**Fig. 2:**
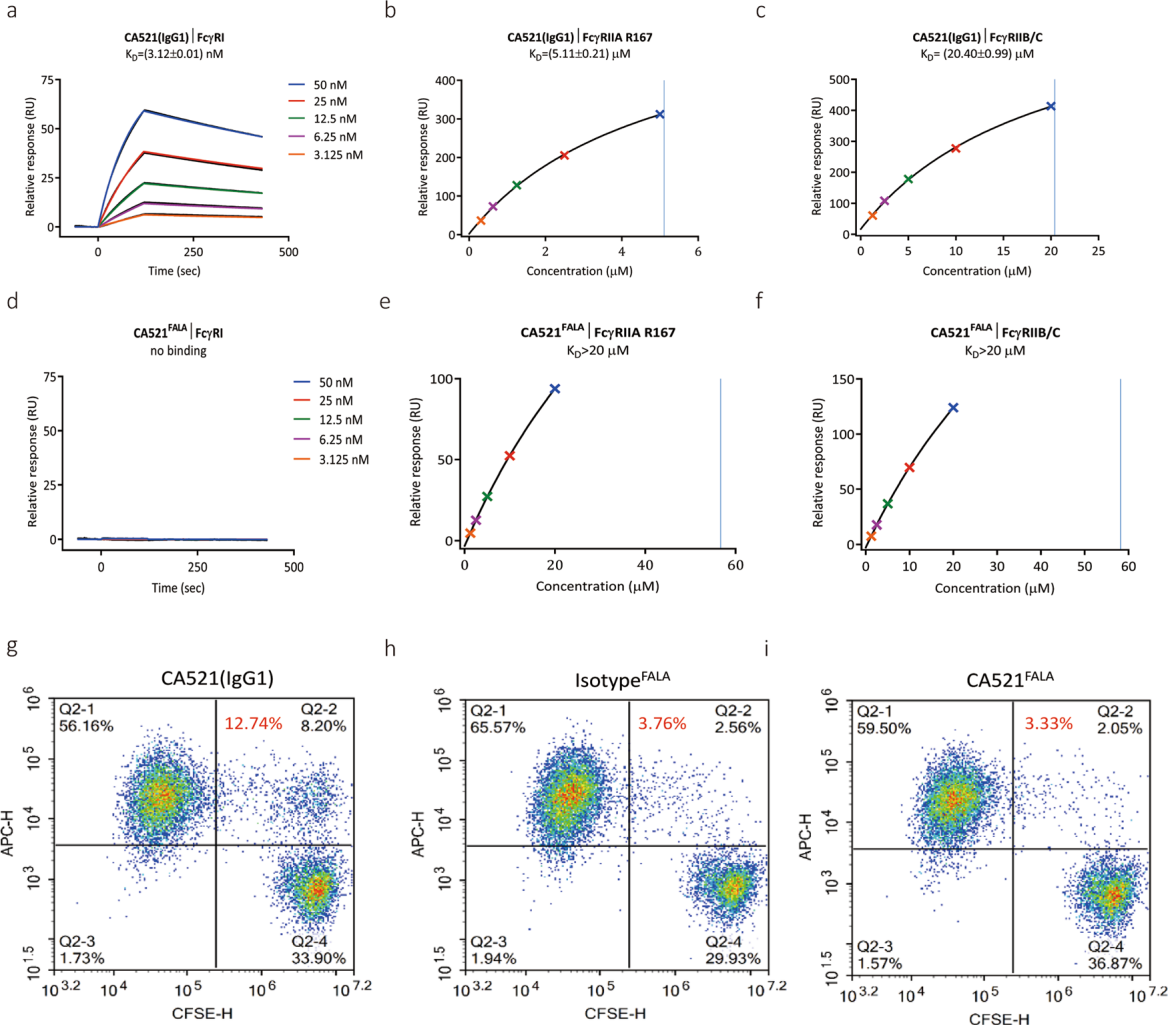
CA521^FALA^ shows modified affinity to various Fc or complement receptors. **a**–**f** Affinity of CA521(IgG1) and CA521^**FALA**^ to FcγRI, FcγRIIA R167, and FcγRIIB/C. It was assessed by the surface plasmon resonance (SPR) assay using the BIAcore 8k system. Experiments were performed in duplicate and one representative data were displayed. **g**–**i** ADCP analysis of CA521 ^FALA^. Target cells were stained with CFSE and macrophages were stained with APC-anti-CD206. Double stained macrophages are considered to have phagocytized target cells. Phagocytosis rate (in red font) = Q2-2%/(Q2-1% + Q2-2%)*100% (double-stained macrophages/APC-anti-CD206 stained macrophages). Phagocytosis rates for CA521(IgG1), isotype, and CA521^FALA^ were 12.74, 3.76, and 3.33%, respectively. Experiments were performed two times and one representative data was displayed.

As mentioned previously, ADE is a potential risk for neutralizing MAbs against SARS-CoV infection. The FALA mutation was introduced to CA521 potentially abrogates the binding with Fc receptors or complement receptors. To confirm the result of the mutation, the binding affinity of CA521^FALA^ and CA521(IgG1) to various human Fc or complement receptors were examined by surface plasmon resonance (SPR) or Elisa assay. The binding affinity of CA521^FALA^ with Gama Fc Receptor 1 (CD64) was below the detection limit and in the case of FcγRIIA R167 and FcγRIIB/C, it was >20 μM (Fig. 2a–f). The affinity of CA521^FALA^ to C1q was also reduced compared with CA521(IgG1) and slightly reduced compared with Opdivo (IgG4) (Supplementary Fig. 6).

To further confirm abrogated binding in a cell-based assay, Antibody-dependent cellular phagocytosis (ADCP) was conducted with SARS-CoV-2 Spike protein transfected CHO-K1 cells as target cells and macrophages derived from CD14^+^ monocytes as effector cells. Target cells were stained with CFSE and macrophages were stained with APC-anti-CD206. Double stained macrophages were considered to be macrophages that have phagocytized target cells. The phagocytosis rate of CA521^FALA^ was similar to the isotype control and lower than that of CA521 (IgG1), like 3.33%, 3.76%, and 12.74% respectively (Fig. 2g–i). For CA521^FALA^, phagocytosis by macrophages was almost completely avoided consistently with the notation that the binding of CA521^FALA^ with Fc receptors was almost abrogated.

### CA521^FALA^ inhibited SARS-CoV-2 infection in vitro and in vivo

The in vitro neutralization abilities of CA521^FALA^ against SARS-CoV-2 infection were evaluated using a pseudoviruses system expressing Spike protein of SARS-CoV-2 and plaque-reduction neutralization test (PRNT) against an authentic SARS-CoV-2 infection of Vero cells.

**Fig. 3:**
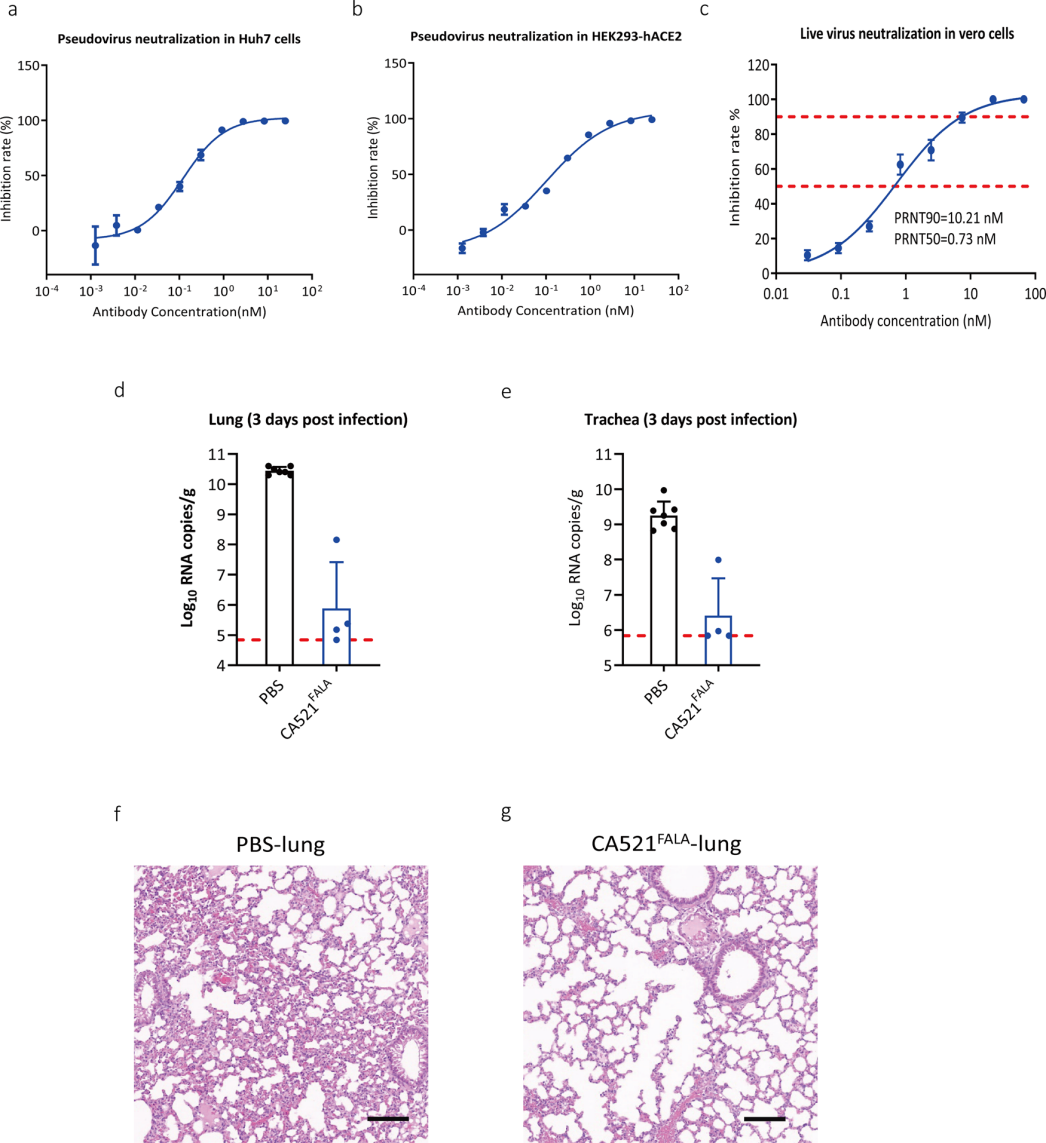
CA521^FALA^ inhibited SARS-CoV-2 infection in vitro and in vivo. **a** CA521^FALA^ inhibits SARS-CoV-2 pseudovirus infection into Huh7 cells. **b** CA521^FALA^ inhibits SARS-CoV-2 pseudovirus infection into hACE2 expressing HEK293T cells. **c** CA521^FALA^ inhibits an authentic SARS-CoV-2 strain (BetaCoV/Beijing/IMEBJ01/2020) infection into Vero cells in vitro. Neutralizing activity of mAbs was measured using a standard plaque reduction neutralization with Vero cells. PRNT50 values were determined using non-linear regression analysis. **d**, **e** CA521^FALA^ exited therapeutic efficacy in SARS-CoV-2 susceptible mice. BALB/c mice who received a SARS-CoV-2 mouse-adapted strain (MASCp6) challenge were administered intraperitoneally with a single dose of 20 mg/kg of CA521 ^FALA^ (*n* = 4) or PBS (*n* = 6) in a therapeutic setting. The level of viral RNA was detected in the lung (**d**) and trachea (**e**) at 3 days post infection (3dpi) with a Quantitative PCR assay. **f**, **g** Histopathological analysis of lung samples from PBS group or CA521 ^FALA^ group at 3 dpi. Scale bar: 100 μm.

CA521^FALA^ can inhibit pseudoviruses transduction into Huh-7 and hACE2 expressing HEK293T cells with IC50 at 0.121 and 0.104 nM, respectively (Fig. 3a, b). In addition, CA521^FALA^ also exhibited strong neutralizing activity against an authentic SARS-CoV-2 strain with a PRNT50 of 0.73 nM (Fig. 3c).

Next, we sought to assess the correlation between in vitro neutralization and in vivo protection. To evaluate the protective efficacy of CA521^FALA^, BALB/c mice who received MAScp6 challenge were administered intraperitoneally with a single dose of 20 mg/kg of CA521 ^FALA^ (*n* = 4) or phosphate-buffered saline (PBS) (*n* = 6) in a therapeutic setting (Fig. 3d–g). As expected, a high level of viral RNAs was detected in the lung and trachea at 3 days post infection in mice who received PBS treatment (Fig. 3d, e). Remarkably, a single dose administration of CA521^FALA^ dramatically reduced RNA viral loads (Fig. 3d, e). CA521^FALA^ treatment resulted in a 34914-fold and 693-fold reduction of viral titers in the lungs and tracheas at 3 dpi, respectively (Fig. 3d, e). Histopathological examination revealed interstitial pneumonia, characterized by inflammatory cell infiltration, alveolar septal thickening, and distinctive vascular system injury developed in BALB/C mice belonging to the PBS control group at 3 dpi (Fig. 3f). In contrast, the lungs in mice from the CA521^FALA^ treated group only showed very mild inflammatory cell infiltration, and no obvious lesions of alveolar epithelial cells or focal hemorrhage (Fig. 3g). These results demonstrate that CA521^FALA^ is a potent neutralizing antibody, which is effective in conferring protection on mice against SARS-CoV-2.

### CA521^FALA^ is stable and has a long half-life in mice and rhesus monkeys

**Fig. 4:**
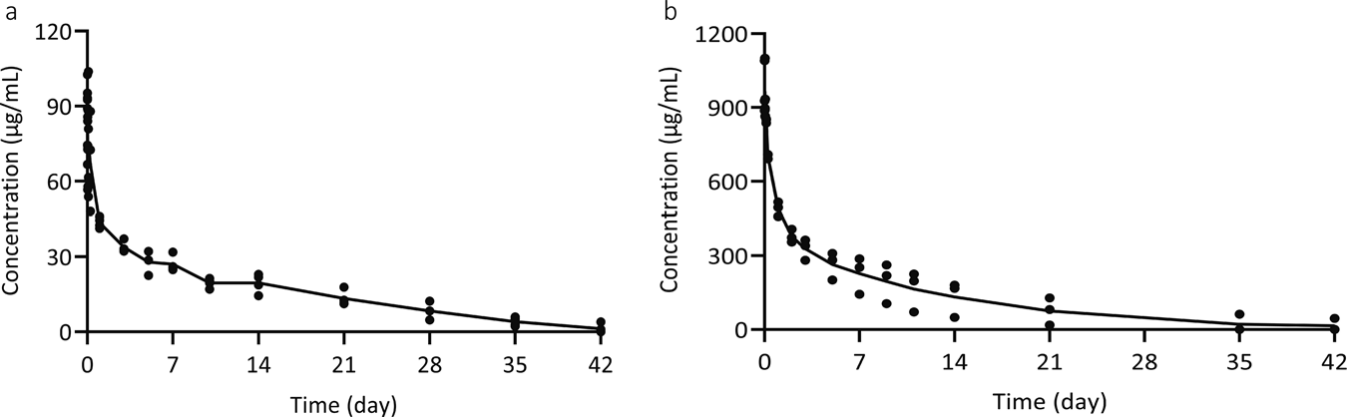
Serum concentration vs. time profiles of CA521^FALA^ in mice and rhesus monkeys. **a** Four mice were administered intravenously at a dose of 10 mg/kg with CA521^FALA^. Antibody concentrations in serum were determined in Elisa with SARS-CoV-2 (2019-nCoV) spike protein as the capture reagent. **b** Three healthy rhesus monkeys were administered intravenously at a dose of 50 mg/kg with CA521^FALA^. The antibody concentration in serum at different time points were determined in Elisa with SARS-CoV-2 (2019-nCoV) spike protein as the capture reagent. The main PK kinetic parameters were calculated using Phoenix WinNonlin.

The pharmacokinetics of the human antibody CA521^FALA^ was studied in mice and rhesus monkeys. To achieve this a single intravenous injection dose of CA521^FALA^ was given to C57BL/6 mice (*N* = 4) and rhesus monkeys (*N* = 3) at 10 and 50 mg/kg. ELISA was used to determine the concentration of CA521^FALA^ in serum. Following a single-dose 10 mg/kg intravascular injection in C57BL/6 mice, CA521^FALA^ showed a bi-exponential serum concentration–time profile with a short distribution phase followed by a long elimination phase, with a terminal half-life (*t*_1/2_, _*λz*_) of 9.5 ± 4 days, *C*_max_ of 96 ± 5 μg/mL and AUC_(0−*t*)_ of 647 ± 43 day*μg/mL(Fig. 4a). Following a single-dose 50 mg/kg intravascular injection in rhesus monkeys, CA521^FALA^ showed a bi-exponential serum concentration–time profile with a short distribution phase followed by a long elimination phase, with a terminal half-life (*t*_1/2, λz_) of 9.3 ± 5 days, *C*_max_ of 975 ± 110 μg/mL and AUC_(0-*t*)_ of 5101 ± 2020 day*μg/mL (Fig. 4b).

### Cryo-electron microscopy analysis of the SARS-CoV-2 Spike protein–CA521^FALA^ complex

**Fig. 5:**
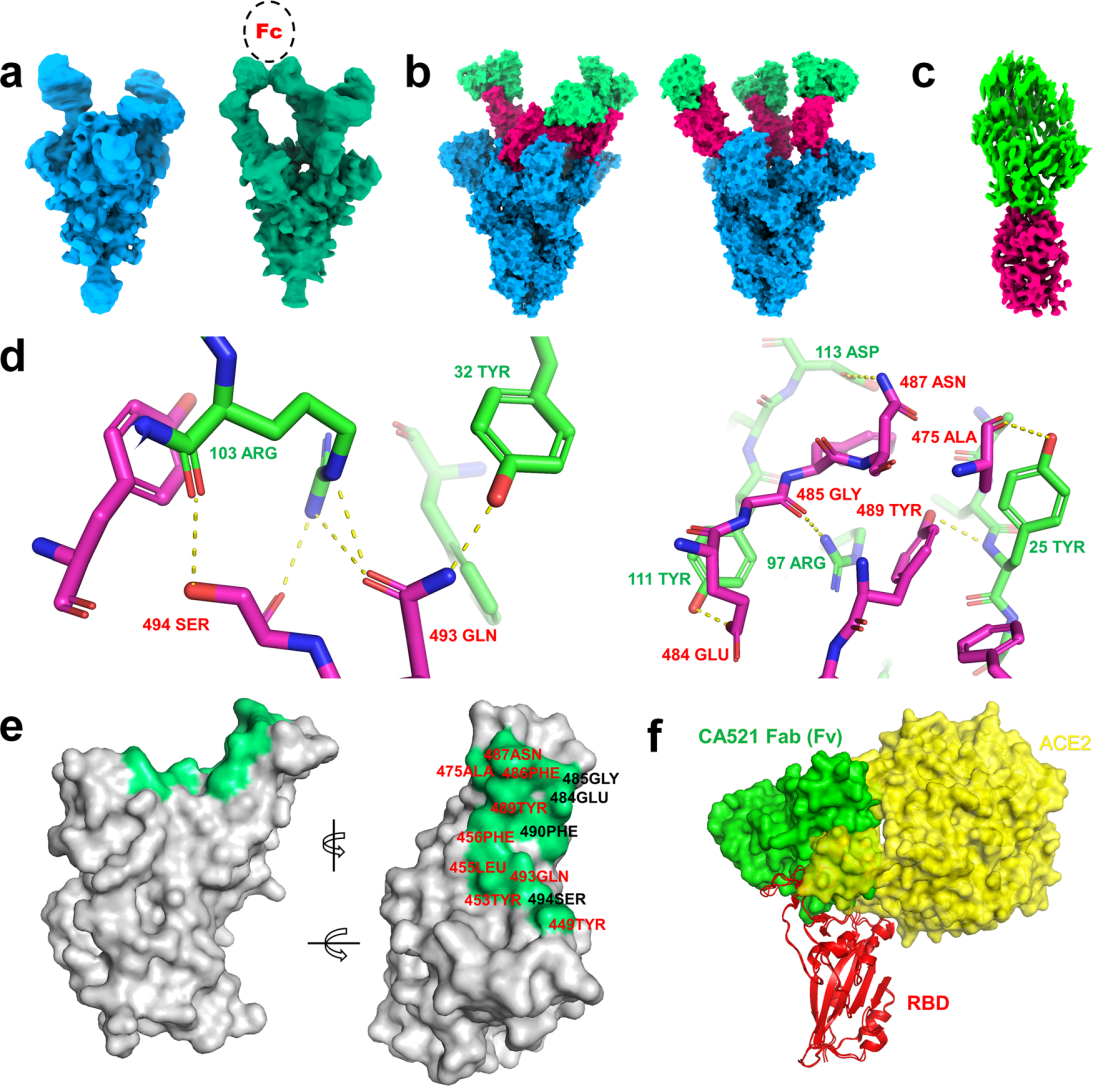
The cryo-EM structures of CA521^FALA^ IgG or Fab in complex with Spike protein. **a** The consensus maps of S-CA521^FALA^ Fab (Left) and S-CA521^FALA^ IgG (Right) low-passed to 10 Å to display the densities of three RBDs and antibodies. The black circle with a dashed line represents the Fc fragment of CA521^FALA^ supposed to connect with two proximal Fabs. All three RBDs are in an open state in the S-CA521^FALA^ IgG complex, while there is one RBD in the closed state in the S-CA521^FALA^ Fab complex. **b** The models of S-CA521^FALA^ Fab (Left) and S-CA521^FALA^ IgG (Right) showed as surface. Only Fv (green) of CA521^FALA^ is well resolved, so other regions are omitted from the models. Magenta: RBD, Blue: another part of Spike protein. **c** The map of one RBD-Fab sub-complex from S-CA521^FALA^ Fab complex after focused refinement. Magenta: RBD, Green: CA521^FALA^ Fab. **d** The polar interactions (dashed yellow lines) between CA521^FALA^ Fab (green) and RBD (magenta). Residues labeled as red are from RBD, residues labeled as green are from Fab. **e** The epitopes of CA521^FALA^ Fab (with a 4 Å cutoff of distance) on RBD (gray surface). The locations of epitopes are displayed as green, and epitopes overlapped with that of ACE2 are highlighted as red labels. **f** CA521^FALA^ Fab (green surface) clashes with ACE2 (yellow surface) when binding to RBD (red cartoon).

To investigate the interaction between CA521^FALA^ and the SARS-CoV-2 Spike protein we determined the structure of this complex using cryo-EM. Details of sample preparation, data collection, and EM analysis are in the methods and extended data (Supplementary Table 3). In brief, the purified extracellular domain of S protein (S-ECD) was incubated with full-length CA521^FALA^ IgG or CA521^FALA^ Fab and then the mixture was applied to prepare cryo-EM grids. After 2D classification, good particles were selected out and then subjected to 3D classification (Supplementary Fig. 7). There was a relatively good class corresponding to 614,999 particles for the S-CA521^FALA^ IgG complex. Particles in this class were subjected to further 3D refinement with C1 symmetry to generate the consensus map of the complex. Then we solved the structure of spike protein in complex with full-length CA521^FALA^ IgG and got a 3.8 Å consensus map. In this structure, all three RBDs were “up” with three Fabs bound asymmetrically (Fig. 5a, b right panel). Two of three Fabs contacted with each other, which suggests the bivalent binding of one CA521^FALA^ IgG molecule with two RBDs. However, in the consensus map the quality of RBD–Fab region was poor, therefore focused 3D classification and refinement of the RBD-Fab region was performed to improve the local resolution of RBD–Fab interface. Unfortunately, the final reconstruction was still not good enough to allow us to identify the epitopes of CA521^FALA^ accurately. Therefore, we replaced full-length IgG with Fab fragment when preparing cryo-EM samples and then repeated the whole structure determination process. Surprisingly the conformation of the S-CA521^FALA^ Fab complex was different from that of the S-CA521^FALA^ IgG complex. Two RBDs in the S-CA521^FALA^ Fab complex were “up” while another one was ‘down’, and all three RBDs were bound with Fab (Fig. 5a, b left panel). The focused refinement on one RBD–Fab sub-complex succeeded to improve the resolution of the RBD–Fab interface to nearly 3 Å (Fig. 5c). At this resolution, the details of the interface between CA521^FALA^ and RBD were unveiled clearly. Hydrogen bonds and salt bridges around RBD residues A475, E484, G485, N487, Y489, Q493, and S494 (Fig. 5d) contributed to the interaction between RBD and CA521^FALA^ together with the hydrophobic interactions around Y449, Y453, L455, F456, F486, and F490. Nine of these 13 residues were overlapped with the ACE2 binding site, including Y449, Y453, L455, F456, A475, F486, N487, Y489, and Q493. The other four CA521^FALA^ interaction residues on the SARS-CoV-2 RBDs are no more than two amino acids away from the direct contact residues of ACE2 on RBD, such as E484, G485, F490, and S494 (Fig. 5e). Superimposition of RBD-CA521^FALA^ Fab and crystal structure of RBD-ACE2 showed that CA521^FALA^ would clash with ACE2 (Fig. 5f), so mAb CA521^FALA^ may block the interaction of SARS-CoV-2 with human ACE2 by occupying the binding site directly. To compare the binding mode of CA521^FALA^ and other antibodies, we mapped their epitopes to the RBD sequences (Supplementary Fig. 8). The epitope of CA521^FALA^ was different from CB6 and H014.

CA521^FALA^ can bind all three RBDs of one SARS CoV-2 spike trimer simultaneously, one IgG binding with two adjacent RBDs in one Spike trimer, and most of its interaction residues with RBD were overlapped with the ACE2-binding site. All these characteristics contribute to the potent blocking and neutralization ability of CA521^FALA^.

## Discussion

There is an urgent need for therapeutic interventions to combat the COVID-19 caused by SARS-CoV-2. Neutralizing antibodies are an important tool that can effectively fight coronavirus^1^. CA521^FALA^ is a human neutralizing antibody that can effectively inhibit pseudovirus and authentic virus infection in vitro by interfering with the mechanism that the virus attaches to the host cell. CA521^FALA^ shows therapeutic efficacy in mice with mouse-adapted SARS-CoV-2 challenge. CA521^FALA^ treatment resulted in 34914-fold and 693-fold reduction of viral titers in the lungs and tracheas at 3 dpi, respectively. Histopathological examination also showed clear improvement in the lung.

CA521^FALA^ overcomes the potential risk of ADE for antibody therapeutics against SARS-CoV-2 infection. By introducing IgG4 subtype and FALA mutation the affinity of CA521^FALA^ to Fc Receptors, C1q, or phagocytosis induced by macrophages for CA521^FALA^ was modified. Furthermore, pharmacokinetic analysis performed in mice and rhesus monkeys revealed that CA521^FALA^ was very stable and had a half-life of 9.5 ± 4 days in mice and 9.3 ± 5 days in rhesus monkeys.

The 3D structures derived from Spike-CA521^FALA^ Fab or IgG complex show that all three RBDs of the Spike protein trimers bound with antibody regardless of the conformation of RBD (Fig. 5a, b). Interestingly, two RBDs were “up” while another one was “down” in the S-CA521^FALA^ Fab complex, but all three RBDs were “up” in the S-CA521^FALA^ IgG complex. The structure of the Spike trimer is similar to previous structures of the SARS-CoV-2 Spike trimer in which there are always some RBDs in the closed state. Recent research shows that ACE2 binding induces the transition of RBD from close to open conformation^15^. CA521^FALA^ IgG may use a mechanism similar to ACE2 and promote the opening of other closed RBDs to facilitate the binding of CA521^FALA^ after binding the “open” RBD.

Up to now most structural studies on neutralizing antibodies of SARS-CoV-2 were based on Fab fragment (including structures solved by both cryo-EM and X-ray), albeit the antibodies COVID-19 patients would inject should be full-length IgG instead of Fab. So there was concern that previously published structures of Spike–Fab complexes may not represent the situations in vivo. Our study displayed the dramatic discrepancy between spike protein bound with full-length IgG and Fab fragment of CA521^FALA^, emphasizing the necessity of studying the full-length form of neutralizing antibodies.

In summary, our study identifies CA521^FALA^ as an excellent neutralizing antibody against SARS-CoV-2 with three major advantages: direct competitive binding with ACE2, binding all three RBDs of one spike simultaneously and bivalent binding of one IgG. CA521^FALA^ shows promise as an effective intervention to the COVID-19 pandemic caused by SARS-CoV-2. Potent neutralizing ability, the low risk of ADE, and long half-life make it an ideal candidate to move on to clinical trial.

## Methods

### Ethical statement

All animal experiments complied with relevant ethical regulations regarding animal research. Pharmacokinetics study protocols in monkeys were approved by Institutional Animal Care and Use Committee(IACUC) and the Approval Number is IACUC-A2020131-K001-01. The animal experiments for in vivo efficacy studies were conducted in the Academy of Military Medical Sciences and approved by the Experimental Animal Committee of Laboratory Animal Center, AMMS (approval no. IACUC-DWZX-2020-001). Protocols of mice experiments for immunization and pharmacokinetics study were approved by LUYE PHARMA Animal Experimentation.

### Regents, mice, cells, and viruses

Reagents, cell lines, and viral strains used in this study are listed in Supplementary Table 2.

### Immunization

Human antibody transgenic mice were generated by Boan Biotechnology. Mice were bred and kept under specific-pathogen-free conditions.

Three mice (named Q1–Q3) were immunized in 10-day intervals with the mixture of recombinant RBD protein (Cat 40592-V05H, Sino Biological), Spike S1 protein (Cat 40591-V02H, Sino Biological) and Spike S2 protein (Cat 40590-V08B, Sino Biological) and boosted by 105 μg mixtures (35 μg for each protein) after three rounds immunization. Two mice (named Q6 and Q7) were immunized in 10-day intervals with recombinant Spike S1 + S2 (Cat 40589-V08B1, Sino Biological) and boosted by 40 μg recombinant Spike S1 + S2 after 3 rounds of immunization. Another two mice (named Q14, Q15) were immunized in 10-day intervals with mixtures of Spike S1 protein (Cat 40591-V02H, Sino Biological) and RBD protein (Cat 40592-V05H, Sino Biological) and boosted by 70 μg mixtures (35 μg for each protein) after three rounds immunization. Genes of these proteins were derived from the SARS-CoV-2 strain (Wuhan-1 strain, GenBank: MN_908947). The antibody titers were tested by Elisa. After three rounds of immunization and one boost, spleen cells were harvested after 3 days of the last boost for phage libraries construction. Supplementary Table 1 showed details for the immunization process and Supplementary Fig. 2 showed serum antibody titers after three rounds of immunization.

### Serum antibody titer detection by ELISA after three rounds of immunization

The titer of serum total anti-Spike antibodies was assessed on serum samples from the orbit vein of mice by ELISA. Blood samples, collected from the mice via orbit vein bleeding at seven days after three rounds immunization, were placed at room temperature for 1 h. After centrifugation at 6000 rpm for 5 min at 4 °C, supernatants were used for ELISA.

High binding ELISA plates were coated with 1 μg/mL recombinant SARS-CoV-2 Spike protein (Sino Biological, 40589-V08B1) for mouse Q1, Q2, Q3, Q6, and Q7 or S1 protein (Sino Biological, 40591-V02H) for mouse Q14 and Q15 at 4 °C overnight, and then blocked with 3% skim milk powder in PBST (PBS containing 0.05% Tween-20) at 37 °C for 1 h. Serially diluted serum (1:100, 1:500, 1:2500, and 1:12500, respectively) in PBST was added into the plates and incubated for 1 h at 37 °C. After washing two times, antibodies binding to coated proteins were detected by peroxidase Labeled Goat anti-human IgG (H + L) (KPL, 474-1006) for Q1, Q2, Q3, Q6, and Q7 or HRP-Goat anti-human Ig Fab (Southern Biotech) for Q14 and Q15. OD450 was read at Multimode Microplate Reader (Thermo Scientific varioskan flash).

### Phage display library construction

RNA was extracted from spleen cells of immunized mouse Q1, Q2, Q3, Q6, and Q7, Q14, and Q15 by Trizol method separately. cDNA synthesis was performed using Transcriptor First Strang cDNA Synthesis Kit. The construct of the phage library was carried out according to the method described in *Phage Display: A Laboratory Manual*^38^.

The variable regions of the heavy and light chains were amplified from the cDNA of each mouse by PCR and then the overlap extension PCR method was used to obtain the ScFvs from variable regions of the heavy and light chains. ScFvs were digested and ligated into pCOMB3x vector, and then these ligated products were electro-transfected into *Escherichia coli* TG1. Helper phage VCSM13 was added for phage amplification. After overnight culture, the supernatant was collected and concentrated into a phage library. The libraries PLQ1, PLQ2, PLQ3, PLQ6, and PLQ7 were derived from mouse Q1, Q2, Q3, Q6, and Q7, respectively. The library PLQ1415 was derived from mouse Q14 and Q15.

### Panning of phage libraries

Recombinant Spike RBD protein was used for panning of libraries PLQ1, PLQ2, PLQ3, and PLQ1415 separately. Recombinant Spike S1 protein was also used for panning of libraries PLQ1, PLQ2, PLQ3, and PLQ1415 separately. Recombinant Spike S1 + S2 protein was used for panning of libraries PLQ6 and PLQ7 separately. Plates coated with 5 μg/mL protein in CBS buffer (15 mM Na_2_CO_3_ and 35 mM NaHCO_3_, pH 9.6) or 20 μL streptavidin-magnetic beads loading 5 μg biotinylated protein were used to capture phages with interest ScFvs. First plates were incubated with input phages at 37 °C for 2 h and beads were incubated at room temperature for 1 h. After washing 5–6 times with PBST, captured phages were eluted by elution buffer (0.1 M HCl-Gly, pH 2.1), neutralized by 1 M Tris buffer (pH 8.0), and then used to infect 5 mL *E. coli* TG1 at 37 °C for 30 min. Helper phage VCSM13 was added for phage amplification. After overnight culture, the supernatant was collected and concentrated into a phage library for the next round of panning. After three rounds of panning, TG1 cells infected eluted phages were grown on plates. ScFvs were expressed and their binding and blocking activity was tested by ELISA. Positive hits were obtained and sequenced.

### Verification of lead candidate CA521^FALA^

#### ELISA-based receptor-binding inhibition assay for screening at Supplementary Fig. 3a

High binding ELISA plates were coated with 0.5 μg/mL recombinant SARS-CoV-2 RBD protein at 4 °C overnight and then blocked with 3% skim milk powder in PBST (PBS containing 0.05% Tween-20) at 37 °C for 1 h. A 4 μg/mL antibody was mixed with 0.04 μg/mL (final concentration) biotinylated human ACE2 and then the mixture was incubated with coated RBD in the plates for 1 h at 37 °C. After washing two times, the retained biotinylated ACE2 binding to coated RBD was detected by HRP-conjugated streptomycin. Inhibition rate % = (OD450 of no antibody − OD450)/OD450 of no antibody * 100%.

#### Pseudovirus neutralization assay-preliminary screening at Supplementary Fig. 3b

SARS-CoV-2 S pseudotyped viruses (using HIV backbone) were provided by Sino Biological Inc. 293T cells overexpressing ACE2 were seeded in 96-well plates (Costar, 3955) at 3 × 10^4^ cells/well. Fifty microlitre serially diluted samples or control mAb (SinoBiological, 40150-D001) were mixed with 50 μL pseudovirus and then they were added into plates seeded with 293T-ACE2 cells (1 × 10^4^ TCID50/mL). Positive control was set up using a mixture of 50 μL DMEM medium and 50 μL pseudovirus. 100 μL DMEM was used as a negative control. Then cells were cultured at 37 °C for 64 h. Luminous value was detected by Luminometer (Berthold Technologies, Centro LB 960) and the inhibitory rate was calculated by (1 − (mean RLU of sample − RLU of negative control)/(RLU of positive control − RLU of negative control)*100%). Experiments were performed in duplicate, value = mean ± SD.

#### Neutralization assay for pseudoviruses-secondary screening at Supplementary Fig. 3d

Pseudoviruses (Cat. 80033) purchased from Beijing SanYao Science & Technology Development Company were produced and titrated as described previously^39^.

SARS-CoV-2 pseudovirus (1.0–2.0 × 10^4^ TCID50/mL) were incubated with threefold serially diluted candidate antibodies at 37 °C for 1 h, and then cell suspensions of Huh-7 (Japanese Collection of Research Bioresources [JCRB], 0403) were added to the mixtures. After 24 h incubation at 37 °C, neutralization potencies of mAbs were evaluated in a luciferase assay. Luciferase activity was measured using Bio-Glo Assay reagent as a substrate (Promega). The percentage of infectivity was calculated as the ratio of luciferase readout in the presence of mAbs normalized to luciferase readout in the absence of mAb. The half-maximal inhibitory concentrations (IC50) were determined using four-parameter logistic regression (GraphPad Prism). Experiments were performed in duplicate, value = mean ± SD.

#### Antibody expression and purification with 293F expression system

Recombinant plasmids were prepared for antibody production. Antibodies were expressed with a 293F Expression system for 7 days (37 °C and 8% CO_2_ at 125 rpm) and the supernatants were harvested and purified by one step of protein A affinity purification. The concentration was determined by UV280 and production yield was calculated by (mg of purified antibodies)/(L of culture volume).

#### Pharmacokinetics studies

A single intravenous injection dose of candidate antibodies (CA521^FALA^, CA530^FALA^, and CA304^FALA^) was conducted in C57BL/6 mice (*N* = 12, 12 males, 4 males/group, age 5–7 weeks, bodyweight 21–23 g) at 10 mg/kg. Blood samples were collected at predose and 3 min, 15 min, 30 min, 1 h, 2 h, 6 h, 24 h, 72 h, 120 h, 168 h, 240 h, 336 h, 504 h, 672 h, 840 h, and 1008 h postdose from the mice via orbit vein bleeding. After placed at room temperature for 30 min, the blood samples were centrifuged at 13,500 rpm for 10 min at 4 °C, and supernatants were analyzed by ELISA. High binding ELISA plates were coated with 1 μg/mL SARS-CoV-2(2019-nCoV) spike protein (Sino Biological, 40592-V08H) at 4 °C overnight, and then blocked with 1% bovine serum albumin (BSA) in PBS at 37 °C for 1 h, followed by washing five times with PBST. Serially diluted antibodies (for standard curve) and 4 fold or 20 fold dilutions of serum samples were added to the plates and incubated at 37 °C for 1 h, followed by washing five times with PBST. Goat Anti-Human IgG-HRP (Southern Biotech, 2049-05) was used as the secondary antibody. The concentration of antibodies in serum was calculated according to the standard curve. The main PK kinetic parameters were calculated using Phoenix WinNonlin.

### Production of human monoclonal antibody

#### Recombinant vector construction

Recombinant antibody heavy chain variable region and light chain variable region were amplified (2× Phanta Max Master Mix, Vazyme, P515-01) using the positive clones screened from the library as the template. Overlap PCR was conducted to assemble variable regions and signal peptides. Purified gene fragments were separately fused (ClonExpress II One Step Cloning Kit, Vazyme, C112-01) into the linearized pcDNA3.4 vectors with constant regions. The recombinant plasmid was prepared for production.

#### Antibody expression and purification

Candidate antibodies were expressed with Expi-CHO Expression system(gibco) for 12days and the supernatant was harvested and purified by protein A resin (GE healthcare). The antibodies were further purified by Q FF (GE healthcare) and Capto S ImpAct (GE healthcare) sequentially and then changed to buffer containing 10 mM CH_3_COONa·3H_2_O, 30 mM NaCl, 5% sucrose, 0.03% tween-20, pH6.0.

#### ELISA-based receptor-binding inhibition assay

High binding ELISA plates were coated with 0.5 μg/mL recombinant SARS-CoV-2 Spike RBD at 4 °C overnight and then were blocked with 3% skim milk powder in PBST (PBS containing 0.05% Tween-20) at 37 °C for 1 h, following two times washing with PBST. Serially diluted CA521^FALA^ was mixed with 0.04 μg/mL (final concentration) biotinylated recombinant human ACE2 and then was incubated with coated RBD in the plates at 37 °C for 1 h. After washing, the biotinylated ACE2 binding to coated RBD was detected by HRP-conjugated Strep. Inhibition rate % = (OD450 of no antibody − OD450)/OD450 of no antibody*100%. Irrelevant mAb with the same constant region of CA521^FALA^ was used as an isotype. Experiments were performed in triplicate, value = Mean ± SD.

#### Cross-reactivity by ELISA analysis

Recombinant SARS-CoV-2 Spike S1 + S2 protein (40589-V08B1, Sino Biological), SARS-CoV Spike protein (SPN-S52H5, Acro), and MERS-CoV Spike protein (40069-V08B, Sino Biological) were coated on high binding ELISA plates with 0.5 μg/mL at 4 °C overnight. Plates were blocked with 3% skim milk powder in PBST at 37 °C for 1 h and then washed two times with PBST. Serially dilutions of mAbs were added following incubation at 37 °C for 1 h. Plates were washed two times and then HRP-goat anti-human IgG (H + L) mAb was used to detect antibodies binding to the Spikes. Irrelevant mAb with the same constant region of CA521^FALA^ was used as an isotype. Experiments were performed in triplicate, value = Mean ± SD.

#### Cell-based binding for CA521^FALA^

SARS-CoV-2 Spike protein transfected CHO cells (GenScript RD00819) were cultured for two passages and then harvested, followed by washing twice with FACS buffer (0.2% BSA in PBS). Totally, 1 × 10^5^ CHO-SARS-CoV-2-Spike cells were stained with isotype control IgG or CA521^FALA^ at a concentration of 0.74 μg/mL at 4 °C for 1 h. After washing by FACS buffer two times, cells were incubated in dark with 100 μL FITC-anti-human IgG Fc (Biolegend, 409310) in 1 μg/mL at 4 °C for 30 min. Cells were washed twice and then resuspended in 100 μL FACS buffer for analysis by NovoCyte 2060R flow cytometry. Irrelevant mAb with the same constant region of CA521^FALA^ was used as an isotype. Experiments were performed in triplicate and one representative data was displayed.

#### Fortebio analysis of antibody binding to CoV spike RBD

Antibodies to be tested were diluted to the concentration of 4 μg/mL with PBST and then immobilized onto Octet Fab2G biosensors for real-time association and dissociation. After arriving at the Signal Change Threshold 1.1 nm and washed in PBST biosensor tips were immersed into the wells containing RBD protein (40592-V05H, Sino Biological) of serial dilutions and allowed to associate for 200 s followed by a dissociation step of 400 s. KD was calculated using a 1:1 binding model in Octet Data Acquisition 9.0.0.49 (Sartorius AG). Experiments were performed in triplicate.

#### Fortebio analysis of antibody binding to Coronavirus spike S1

Antibodies were diluted to the concentration of 8 μg/mL with PBST and then immobilized onto Octet Fab2G biosensors for real-time association and dissociation. After arriving the Signal Change Threshold 1.1 nm, biosensor tips were immersed into the wells containing S1 protein (40591-V02H, Sino Biological) of serial dilutions and allowed to associate for 100 s followed by a dissociation step of 250 s. KD was calculated using a 1:1 binding model in Octet Data Acquisition 9.0.0.49 (Sartorius AG). Experiments were performed in triplicate.

#### Fortebio analysis of antibody binding to CoV spike Spike-Trimer

Antibodies were diluted to the concentration of 3 μg/mL with PBST and then immobilized onto ProA biosensors. After arriving at the Signal Change Threshold 0.8 nm and washing with PBST, biosensor tips were immersed into the wells containing Spike-Trimer protein of serial dilutions and allowed to associate for 200 s followed by a dissociation step of 400 s. The KD was calculated using a 1:1 binding model in Octet Data Acquisition 9.0.0.49. Experiments were performed in triplicate.

#### Antibody-dependent cellular phagocytosis

ADCP was conducted with SARS-CoV-2 Spike protein transfected CHO-K1 cells as target cells and macrophages derived from CD14+ monocytes as effector cells. CD14+ monocytes were cultured in 1640 medium (Gibco, 61870-036) containing 50 ng/mL GM-CSF(Acro, GMF-H4214) for 3 days. Cells were cultured for another 3 days after the medium was replaced with a fresh 1640 medium containing 50 ng/mL GM-CSF. Then IFN-γ (Acro, IFG-H4211) and LPS (Invitrogen, 00-4976-93) were added to the medium to 20 ng/mL and cells were cultured for 2 days to become mature macrophages. Target cells stained with CFSE (Invitrogen, 65-0850-85), macrophages, and antibody were added into 96-well plates and incubated in cell incubator at 37 °C for 2 h. The ratio of macrophages and target cells was 1:1, the concentration of antibody was 2 μg/mL and 1640 medium was used as a buffer. Cells were washed twice and resuspended by DPBS (BOSTER, PYG0021). FcR blocking reagent (Miltenyi Biotec, 130-059-901) was added in 10 μL/well and incubated for 15 min at 4 °C. Then APC-anti-CD206 (Biolegend, 321110) was added in 2 μL/well, followed by incubation at 4 °C for 30 min. Cells were washed twice and resuspended by 100 μL DPBS for detection at NovoCyte 2060R flow cytometry. Double stained macrophages are considered to have phagocytized target cells. Phagocytosis rate (in red font) = Q2-2%/(Q2-1%+Q2-2%)*100%. Experiments were repeated twice and one result was shown.

#### ELISA-based C1q binding assay

High binding ELISA plates were coated with 100 μL 5 μg/mL various CA521 subtypes or OPDIVO (IgG4) at 4 °C overnight. Plates were washed four times with PBS and blocked with PBST containing 1% BSA at 37 °C for 1.5 h. Fourfold serial dilutions of mAbs starting at 40 µg/ml were added and plates were incubated for 2 h at 37 °C with shaking of 180 rpm. HRP labeled anti-human C1q secondary antibody diluted in 1:300 with PBST was used to detect C1q binding with mAbs.

#### The affinity of CA521 with different subtype to FcγRs

The binding kinetics were assessed by SPR assay using the BIAcore 8 K system. The measured equilibrium constant (KD) Measurements were performed at room temperature with CM5 chip, which was amino coupled by His Capture Kit. HBS-EP + buffer (150 mM NaCl, 10 mM HEPES, 3 mM EDTA and 0.05% (v/v) surfactant P20 pH 7.4) was used as running buffer. The blank channel of the chip served as the negative control. FcγRs were captured on the chip. Twofold serial dilutions of mAbs starting at different concentrations (Supplementary Table 4) were applied to flow over the chip surface which was regenerated with 10 mM glycine–HCl (pH 1.5) after each cycle. The affinity was calculated using a 1:1 (Langmuir) binding fit model with BIA evaluation software.

#### Pharmacokinetics studies of CA521^FALA^ in rhesus monkeys and C57BL/6 mice

A single intravenous injection dose of CA521 was conducted in rhesus monkeys (*N* = 3, 2 females and 1 male, age 3–5 years, bodyweight 3.00–3.90 kg) and C57BL/6 mice (*N* = 4, 4 males, age 5–7 weeks, bodyweight 21–23 g) at 50 and 10 mg/kg. PK samples were collected at predose and 5 min, 30 min, 1 h, 3 h, 6 h, 24 h, 48 h, 72 h, 120 h, 168 h, 216 h, 264 h, 336 h, 504 h, 840 h, 1008 h postdose from money, and at predose and 3 min, 15 min, 30 min, 1 h, 2 h, 6 h, 24 h, 72 h,120 h,168 h, 240 h, 336 h, 504 h, 672 h, 840, 1008 h postdose. ELISA was used to determine the concentration of CA521^FALA^ in serum. In this method, SARS-CoV-2(2019-nCoV) spike protein (Sino Biological, 40592-V08H) was used as the capture reagent, and goat anti-human IgG, monkey ads-HRP was detecting agent. The quantification range of the calibration curve was 78.1–10,000 ng/mL for monkeys. The quantification range of the calibration curve was 39.06–5000 ng/mL for mice. The main PK kinetic parameters were calculated using Phoenix WinNonlin.

#### Pseudoviruses neutralization assay for CA521^FALA^

Pseudoviruses (80033) purchased from Beijing SanYao Science & Technology Development Company were produced and titrated as described previously^39^.

SARS-CoV-2 pseudovirus (2.0–2.6 × 10^4^ TCID50/mL) were incubated with 3-fold serially diluted CA521^FALA^ at 37 °C for 1 h, and then cells suspension of Huh-7 (Japanese Collection of Research Bioresources [JCRB], 0403) or HEK-293T-hACE2(CHENGDU NB BIOLAB CO., LTD.) were added to the mixtures. After 24 h incubation at 37 °C, neutralization potencies of mAbs were evaluated in a luciferase assay. Luciferase activity was measured using Bio-Glo Assay reagent as a substrate (Promega). The percentage of infectivity was calculated as the ratio of luciferase readout in the presence of mAbs normalized to luciferase readout in the absence of mAb. The half-maximal inhibitory concentrations (IC50) were determined using four-parameter logistic regression (GraphPad Prism). Experiments were performed in triplicate.

#### Infectious SARS-CoV-2 neutralization assay

Neutralizing activity of mAbs was measured using a standard plaque reduction neutralization with Vero cells. Briefly, fivefold serial dilutions of mAbs were added to approximately 100 PFU of SARS-CoV-2 and incubated for 1 h at 37 °C. Then, the mixture was added to Vero cell monolayers in a 24-well plate in duplicate and incubated for 1 h at 37 °C. The mixture was removed, and 1 ml of 1.0% (w/v) LMP agarose (Promega) in DMEM plus 4% (v/v) FBS was layered onto the infected cells. After further incubation at 37 °C for 2 days, the wells were stained with 1% (w/v) crystal violet dissolved in 4% (v/v) formaldehyde to visualize the plaques. PRNT50 values were determined using non-linear regression analysis. All experiments were performed followed the standard operating procedures of the approved Biosafety Level-3 facility.

#### Infection and antibody treatment of mice

Experiments involving live SARS-CoV-2 viruses were performed in the enhanced biosafety level 3 (P3+) facilities in the Institute of Microbiology and Epidemiology, Academy of Military Medical Sciences.

Specific pathogen-free 6–8-week female Balb/C mice were lightly anesthetized with isoflurane and transduced intranasally with 6 × 10^3^ PFU of SARS-CoV-2 mouse-adapted strain (MASCp6) in 30 µl DMEM. Groups of Balb/C mice that received SARS-CoV-2 challenge were treated intraperitoneally with a single dose of 20 mg/kg of CA521^FALA^ (*n* = 4) or PBS (*n* = 6) at 2 h post infection. The lung and trachea tissues of mice were collected at 3 dpi for virus titer and autopsy test.

#### Viral RNA quantitation

Viral burden in lung and trachea from mice were measured as described previously. Briefly, lung and trachea tissue homogenates were clarified by centrifugation at 6000 rpm for 6 min and viral RNA was extracted using the QIAamp Viral RNA Mini Kit (Qiagen) according to the manufacturer’s protocol. Viral burden in each tissue sample was performed by quantitative reverse transcription PCR (RT-qPCR) targeting the S gene of SARS-CoV-2. RT-qPCR was performed using the One Step PrimeScript RT-PCR Kit (Takara). The determination of the detection limit was based on the lowest level at which viral RNA was detected and remained within the range of linearity of a standard curve (Ct value of 38). RT-qPCR was performed using One Step PrimeScript RT PCR Kit (Takara, Japan) with the following primers and probes: CoV-F3 (5′-TCCTGGTGATTCTTCTTCAGGT-3′); CoV-R3 (5′-TCTGAGAGAGGGTCAAGTGC-3′); and CoV-P3 (5′-FAM-AGCTGCAGCACCAG CTGTCCA-BHQ1-3′). The 20 μl reaction mixtures were set up with 2 μl of RNA. Cycling conditions were as follows: 42 °C for 5 min, 95 °C for 10 s, followed by 40 cycles of 95 °C for 5 s and 60 °C for 20 s.

#### Histology and immunostaining

Lung tissues were excised and fixed with 10% neutral buffered formalin, dehydrated, and embedded in paraffin. Sections at a thickness of 4 μm were stained with hematoxylin and eosin (H&E) according to standard histological procedures. Images were captured using an Olympus BX51 microscope equipped with a DP72 camera.

#### Spike protein expression and purification

Spike protein used in this study was prepared as previously described. Briefly, the extracellular domain (ECD) (1–1208 aa) of spike protein was cloned into the pCAG vector (Invitrogen) with two proline substitutions at residues 986 and 987, a “GSAS” substitution at residues 682–685 and a C-terminal T4 fibritin trimerization motif followed by one Flag tag and the construct was overexpressed in HEK 293F cells. Spike ECD secreted into the medium was purified by one step of anti-Flag affinity purification and size-exclusion chromatography.

### Cryo-electron microscopy analysis of the SARS-CoV-2 Spike protein-CA521^FALA^ IgG/Fab complex

#### Sample preparation

Totally, 15 µL purified extracellular domain of S protein (S-ECD) at the concentration of 2 mg/mL was incubated with 2.5 µL CA521^FALA^ IgG at the concentration of 6.6 mg/mL or CA521^FALA^ Fab at the concentration of 5.8 mg/mL on ice for 1 min and then the mixture was applied to prepare cryo-EM grids.

For cryo-EM, frozen-hydrated specimens were prepared with a Thermo Fisher Vitrobot Mark IV plunger. Totally, 3.5 μl of S-ECD and CA521^FALA^ IgG or Fab complex was placed on a glow discharged holey carbon grid (Quantifoil Au R1.2/1.3) with or without continuous reduced graphene oxide film (gift from Dr. Nan Liu in Prof. Hongwei Wang’s lab at Tsinghua University). The excess of solution from the grid was blotted for 0.5 s at 100% humidity at 8 °C before the grid was flash-frozen in liquid ethane slush cooled at liquid-nitrogen temperature.

#### Data collection

Cryo-EM data were collected on a Thermo Fisher Titan Krios G3i electron microscope equipped with a Gatan K3 direct electron counting camera. The microscope was operated at 300 kV, and images of the specimen were recorded with a defocus range of −1.4 to −2.4 µm at a calibrated magnification of 64kx in super-resolution mode of the K3 camera, thus yielding a pixel size of 0.54 Å on the object scale. Movie stacks each containing 32 sub-frames were recorded with the semi-automated low-dose acquisition program EPU, with an electron dose rate of 18.6 electrons/Å^2^/s and a total exposure time of 2.6 s.

#### EM analysis

The raw super-solution dose-fractionated image stacks were 2× Fourier binned, aligned, dose-weighted, and summed using MotionCor2, resulting in summed micrographs in a pixel size of 1.087 Å per pixel. Contrast transfer function (CTF) parameters were estimated using CTFFIND4.1. The following processing steps were performed in RELION3.1. The First Laplacian-of-Gaussian method was used to pick particles automatically. Then all these particles were subjected to several rounds of reference-free 2D classification to remove contaminants and bad particles. After that 3D classification was performed using a map derived from the PDB model as the initial reference model. The most homogeneous particles were selected for the final 3D auto-refinement. Reconstruction resolutions were determined based on the gold-standard Fourier shell correlation (FSC) 0.143 criterion with the high-resolution noise substitution.

#### Statistics and reproducibility

All statistical analyses were performed in GraphPad Prism 8 software. Details on the statistical tests applied are provided within the figure legends. The data are reported as bar graphs displaying individual values and Means ± SD, as indicated in the figure legends. No experiments were excluded from the analyses.

## Supplementary information

Supplementary Information

Supplementary Data 1

## Data Availability

The cryo-EM maps have been deposited in EMDB under accession codes EMD-30629 (the consensus map of S-CA521 IgG), EMD-30950 (map of focused refinement on Fab-RBD from S-CA521 Fab complex), and EMD-30951 (the consensus map of S-CA521 Fab). The atomic model of focused refinement on Fab-RBD has been deposited in PDB under accession code 7E23. The sequences of CA521^FALA^ MAbs have been deposited in GenBank with the accession codes MW454370 for the light chain and MW454371 for the heavy chain. The data used to support the findings of this study are included within the article.
