# Supplementary material for: Structure and function analysis of a potent human neutralizing antibody CA521FALA against SARS-CoV-2
Source: Commun Biol. 2021 Apr 23;4:500. doi: 10.1038/s42003-021-02029-w (PMC8065039; doi:10.1038/s42003-021-02029-w)
Supplement: Supplementary file 1 — Supplementary Information [file 42003_2021_2029_MOESM1_ESM.pdf]

Supplementary Materials for

Structure and function analysis of a potent human neutralizing antibody CA521^{FALA} against SARS-CoV-2

Deyong Song[1]*, Wenbo Wang[2]*, Chuangchuang Dong[1], Zhenfei Ning[1], Xiu Liu[1], Chuan Liu[3], Guangying Du[4], Chunjie Sha[4], Kailin Wang[1], Jun Lu[1], Baiping Sun[1], Yanyan Zhao[1], Qiaoping Wang[1], Hongguang Xu[1], Ying Li[1], Zhenduo Shen[1], Jie Jiao[1], Ruiying Wang[4], Jingwei Tian[4], Wanhui Liu[4], Lan Wang[2✉], Yong-Qiang Deng[5✉], Changlin Dou[1✉]

*These authors contributed equally to this work.

Corresponding author:

✉Corresponding author. e-mail: DouChanglin@luye.com (C.D.); dengyq1977@126.com (Y.Q.D.); wanglan@nifdc.org.cn(L.W.)

This PDF file includes:

Supplementary Fig. 1 to Fig. 8

Supplementary Table 1 to 4

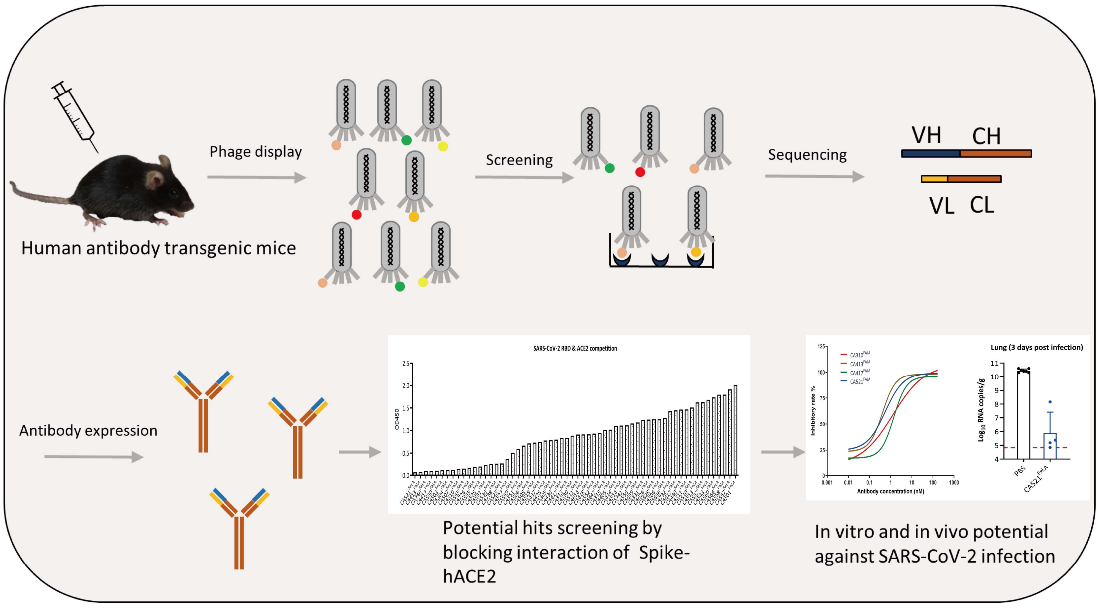

Supplementary Fig. 1 Graphical Abstract for antibody screening. Sixty potential clones with spike-hACE2 blocking activity were obtained through immunizing human antibody transgenic mice followed by phage display. After in vitro and in vivo evaluation, CA521^{FALA} show the best potential against SARS-CoV-2 infection.

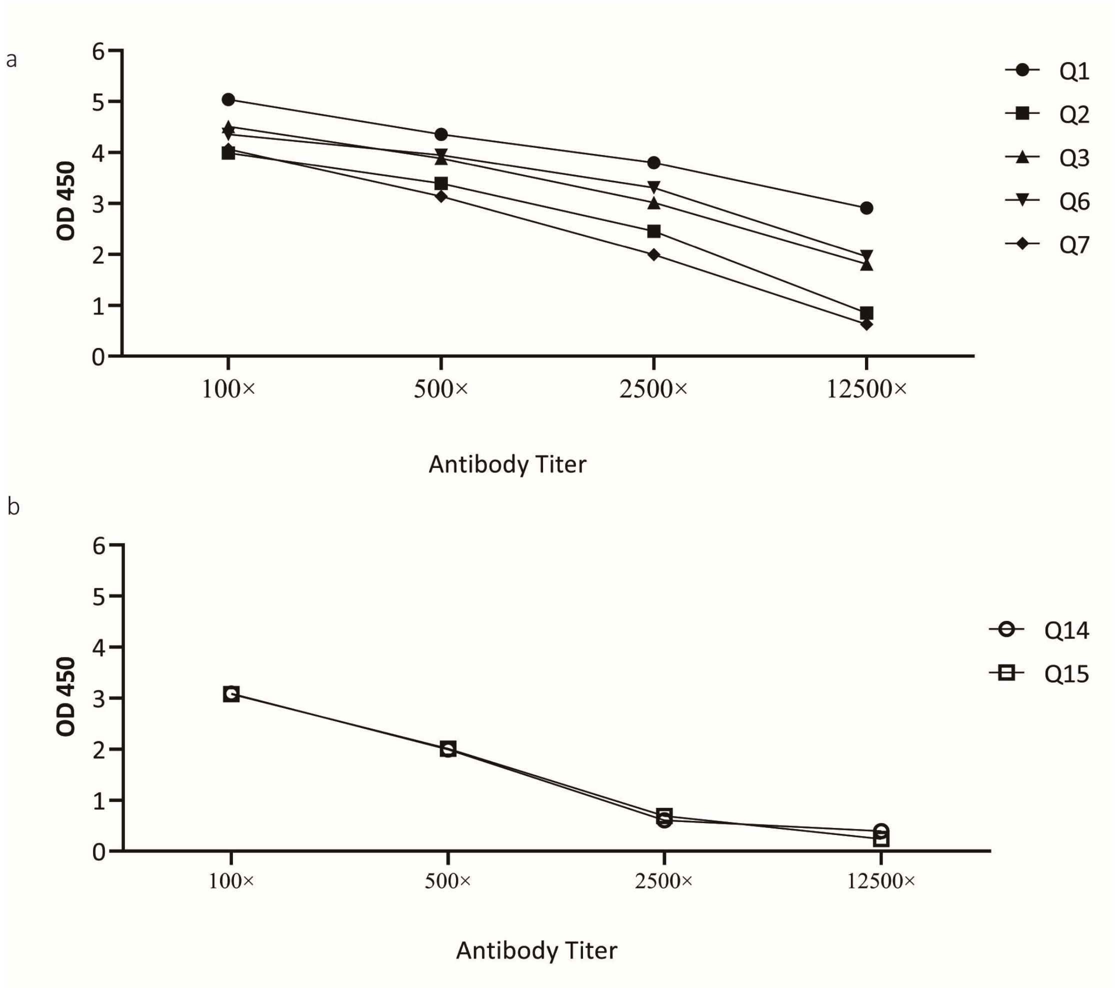

Supplementary Fig. 2 Serum antibody titer detection for 7 mice after 3 rounds immunization. ELISA was used to determine the antibody titer in serum from 7 mice at seven days after 3 rounds immunization. (a) SARS-CoV-2 Spike protein was coated as the capture reagent for serum samples of mouse Q1, Q2, Q3, Q6 and Q7. (b) SARS-CoV-2 S1 protein was coated as the capture reagent for serum samples of mouse Q14 and Q15. OD450 was read at Multimode Microplate Reader. The serum antibody titer was determined by serially diluting serum samples (1:100, 1:500, 1:2500 and 1:12500, respectively).

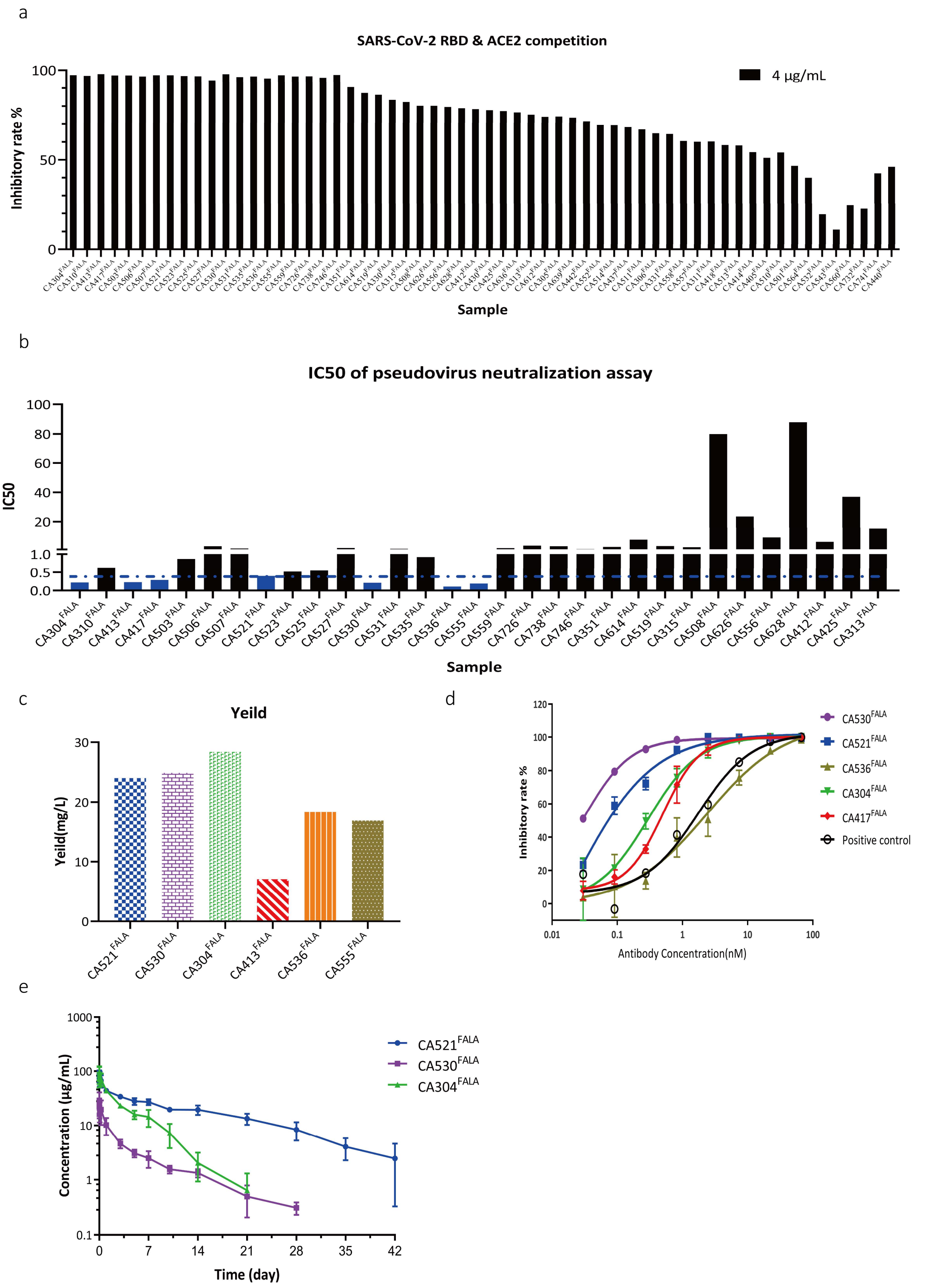

Supplementary Fig. 3 Verification of lead candidate CA521^{FALA}. (a)Blocking activity on ACE2 and RBD interaction for sixty antibodies were analyzed by ELISA. Thirty-four candidates with better blocking activity were selected for neutralization activity evaluation. (b) 7 candidates showed lower IC50 (blue) in neutralization assay for pseudoviruses with 293T-ACE2 as target cells. (c) Production yield for the 7

candidates in 293F expression system. (d)The sequence of CA555^{FALA} was close to CA530^{FALA} and CA413^{FALA} had lower production yield. The left 5 candidates were further evaluated in neutralization assay for pseudoviruses with Huh-7 cells as target cells. (e)Three candidates with better neutralization activity were picked up to carry out pharmacokinetics study in mice. A single dose administration (intravenous) for CA521^{FALA}, CA530^{FALA} and CA304^{FALA} was conducted in mice at 10 mg/kg. ELISA was used to determine the concentration of antibodies in serum.

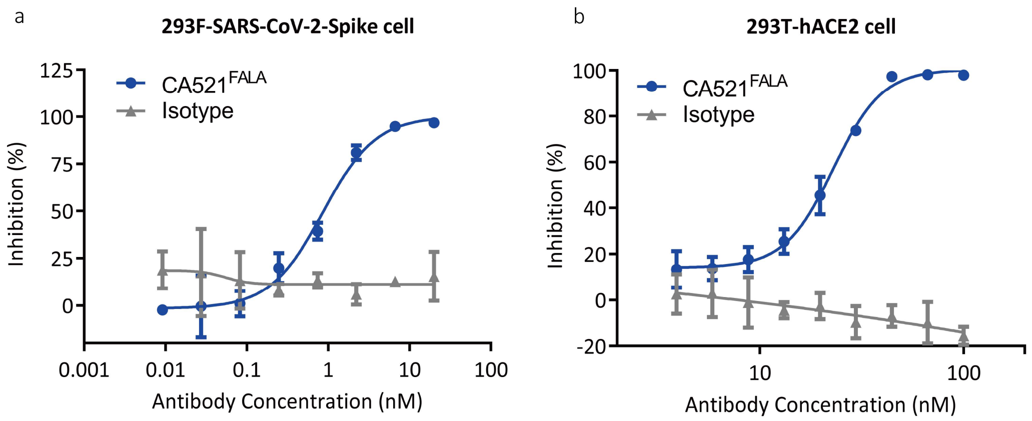

Supplementary Fig. 4 CA521[FALA] blocked the binding of recombinant ACE2 to SARS-CoV-2 Spike expressing 293F cells and the binding of SARS-CoV-2 RBD to ACE2 expressing 293T cells.

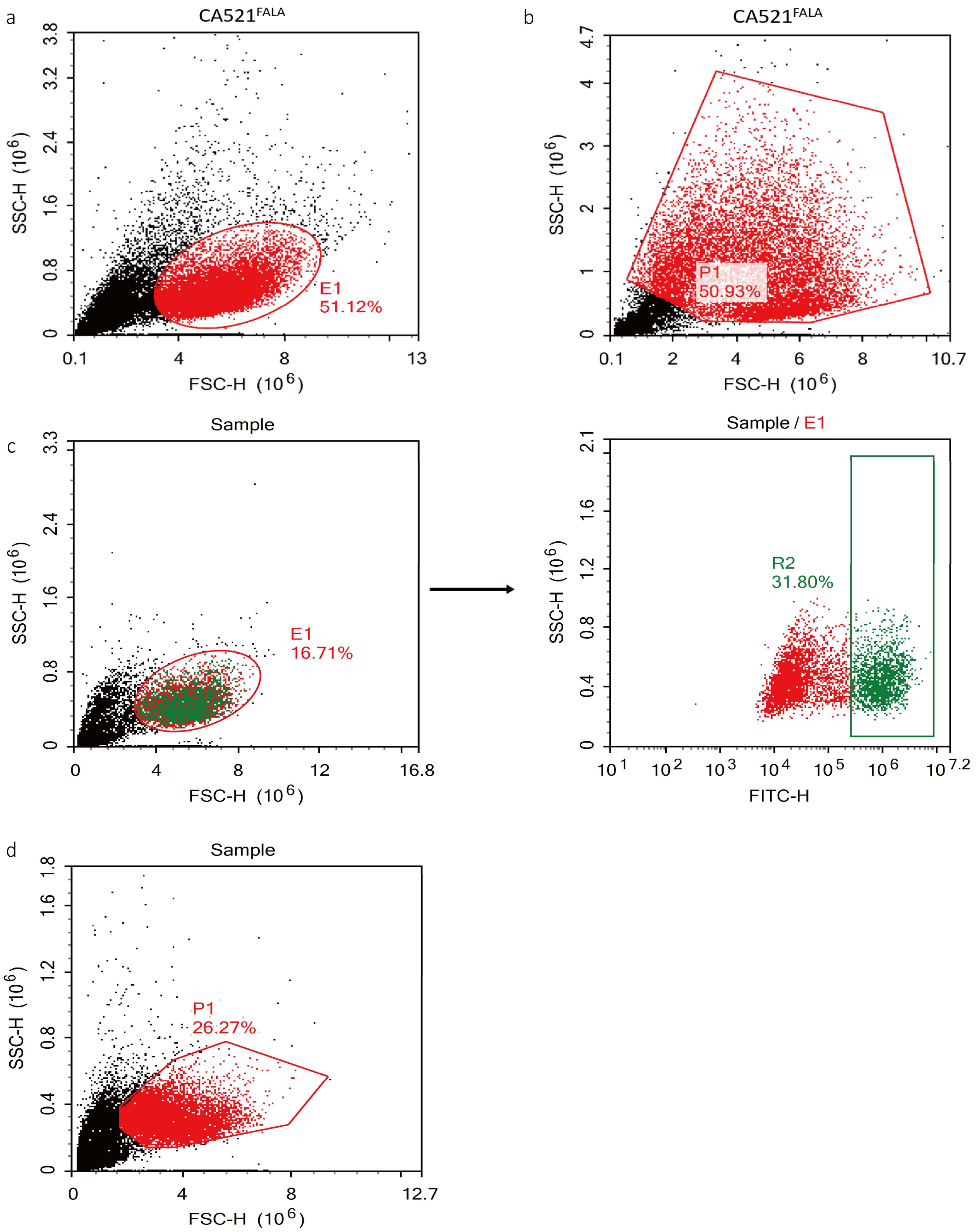

Supplementary Figure 5. Gating strategy for flow cytometry. (a) Gating strategy for cell based binding for CA521^{FALA}. Live cells were gated by FSC-H and SSC-H, and then analyzed by FITC-H and Count. Plots are representative of 3 experimental replicates. (b) Gating strategy for Antibody-Dependent Cellular Phagocytosis assay. Live cells were gated by FSC-H and SSC-H, and then phagocytosis was analyzed by FITC-H and APC-H. Plots are representative of 2 experimental replicates of all samples. (c) Gating strategy for blocking activity of CA521^{FALA} using SARS-CoV-2 Spike expressing 293F cells. After live cells were gated by FSC-H and SSC-H, SARS-CoV-2 Spike positive cells were gated by FITC-H and SSC-H as EGFP was infused with Spike protein. APC-Streptavidin was used to detect cell-binding ACE2-biotin, and then cells

were analyzed by APC-H and Count. Plots are representative of 3 experimental replicates of all samples. (d) Gating strategy for blocking activity of CA521^{FALA} using ACE2 expressing 293T cells. After live cells were gated by FSC-H and SSC-H, they were analyzed by FITC-H and Count as FITC-Streptavidin was used to detect cell-binding Spike RBD-biotin. Plots are representative of 3 experimental replicates of all samples.

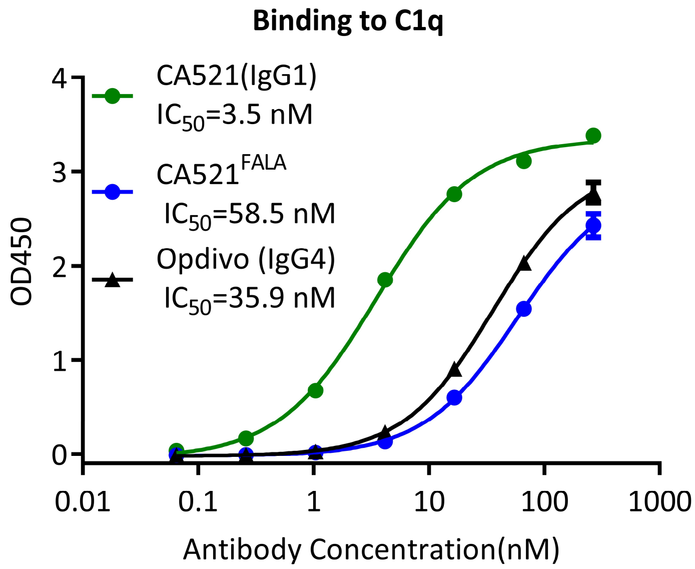

Supplementary Fig. 6 Affinity of CA521^{FALA} for C1q was reduced compared with CA521(IgG1) and slightly reduced compared with Opdivo (IgG4). Affinity for C1q was monitored in Elisa assay. Experiments were performed in duplicate, value = Mean ± SD.

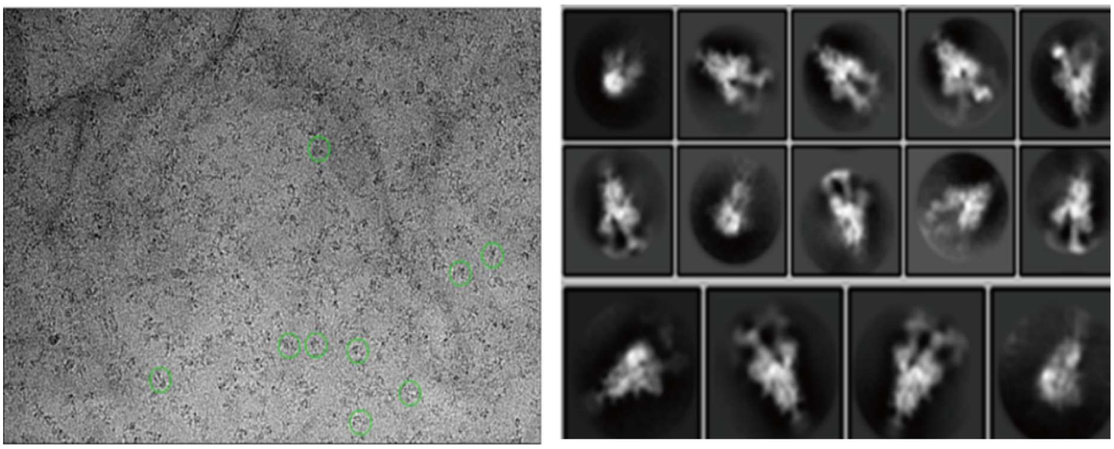

Supplementary Fig. 7 Raw cryo-EM micrograph (Left) and good 2D class averages (Right) of S-CA521^{FALA} IgG complex. The green boxes show the typical particles of S-CA521 IgG complex on reduced graphene oxide film.

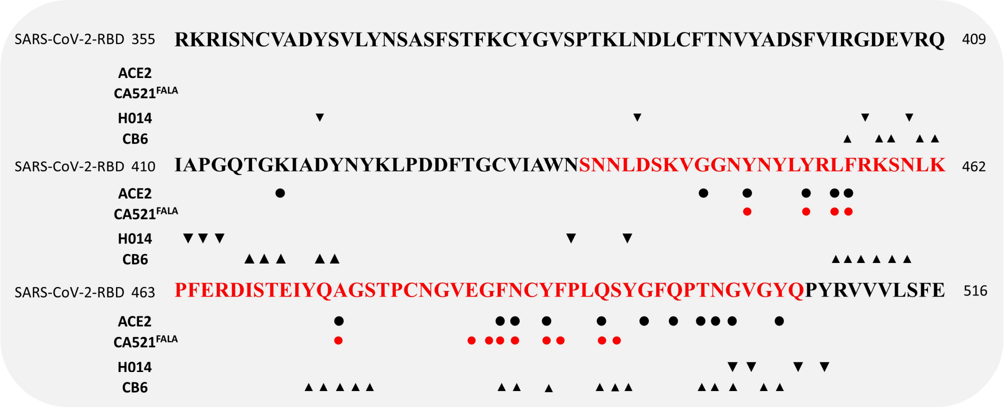

Supplementary Fig. 8 Sequence of the SARS-CoV-2 RBD. The interacting residues for hACE2 in the SARS-CoV-2 RBD are indicated by black dots; binding sites for CA521^{FALA} are indicated by red dots; primary binding sites for H014 are indicated by inverted black triangle; binding sites for CB6 are indicated by black triangle.

Supplementary Table 1. Immunization and panning process.

Mouse ID	Immune antigen	Immune Rounds	Booster	Library name	Protein used for panning	Number of positive clones with different sequences(60 in all)	Candidate antibody
Q1	Mixture of recombinant RBD, Spike S1 and Spike S2 protein	3	35μg RBD, 35μg Spike S1 and 35μg Spike S2 protein	PLQ1	RBD	15	CA304^{FALA},CA413^{FALA}
					Spike S1	4	\
Q2				PLQ2	RBD	8	CA521^{FALA},CA530^{FALA}
					Spike S1	2	\
Q3				PLQ3	RBD	12	\
					Spike S1	0	\
Q6	Recombinant Spike S1+S2(Full lenth)	3	40μg Spike S1+S2(Full lenth) protein	PLQ6	Spike S1+S2(Full lenth)	0	\
Q7				PLQ7		0	\
Q14	Mixture of recombinant Spike S1 and RBD protein	3	35μg Spike S1 and 35μg RBD protein	PLQ1415	RBD	17	\
Q15					Spike S1	2	

Supplementary Table 2. Regents, Mice, Cells and Viruses

REAGENT or RESOURCE	IDENTIFIER	SOURCE
2019-nCoV Spike S1+S2	40589-V08B1	Sino Biological
2019-nCoV Spike RBD	40592-V05H	Sino Biological
2019-nCoV Spike S1	40591-V02H	Sino Biological
2019-nCoV Spike S2	40590-V08B	Sino Biological
MERS-CoV Spike	40069-V08B	Sino Biological
SARS S protein	SPN-S52H5	Acro Biosystems
Transcriptor First Strang cDNA Synthesis Kit	4897030001	Roche
C1q	Ab96363	Abcam
FcγRI	1257-Fc	R&D systems
FcγRIIA R167	1330-CD/CF	R&D systems
FcγRIIB/C	1875-CD	R&D systems
SfiI	R0123L	New England Biolabs
TG1 competent cells	60502-2	Lucigen
PEG8000	A600433-0500	BBI Life Sciences
NaCl	10019318	Sinopharm
Dynabeads M-280 Streptavidin	11206D	Invitrogen
Ez-link Sulfo-NHS-LC-Biotin	21335	Thermo Fisher Scientific
Stripwell Microplate	42592	Costar
Human ACE2	C05Y	Novoprotein
High binding plates	40301	Beaver
Skim milk powder	232100	BD Biosciences
PBS	P1010	Solarbio
Tween-20	T8220	Solarbio
Affinity purified antibody	474-1006	KPL

peroxidase Labeled Goat anti-human IgG(H+L)		
Goat Anti-Human IgG-HRP	2049-05	Southern Biotech
BSA	A1933-100g	Sigma
SureBlue Reserve TMB Microwell Peroxidase Substrate (1-Component)	5120-0081	KPL
Human IgG-heavy and light chain monkey ads-HRP	A80-319P-26	BETHYL
Streptavidin/HRP	890803	R&D systems
96 well Microplate	3955	Costar
Control mAb	40150-D001	SinoBiological
DMEM	Gibco	11965092
FITC-anti human IgG Fc mAb	409310	Biolegend
anti-C1q antibody (HRP)	ab46191	Abcam
CM5 chip	BR-1005-30	GE healthcare
HBS-EP+ buffer	BR-1006-69	GE healthcare
Human antibody capture kit	29-2346-00	GE healthcare
His Capture Kit	28-9950-56	GE healthcare
Fab2G biosensors	18-5125	ForteBio
ProA biosensors	18-5010	ForteBio
CFSE	65-0850-85	Invitrogen
1640	61870-036	Gibco
GM-CSF	GMF-H4214	Acro
IFN-γ	IFG-H4211	Acro

LPS	00-4976-93	Invitrogen
DPBS	PYG0021	BOSTER
FcR Blocking Reagent	130-059-901	Miltenyi Biotec
APC anti-human CD206	321110	BioLegend
CD14+ Monocytes	PB011F-C-1M	AllCells
CHO K1-Spike cell line	RD00819	GenScript
293F-SARS-CoV-2-Spike cells	/	NbBiolab
293T-hACE2 cells	/	NbBiolab
Huh-7 cells	0403	JCRB
VERO CELL	CCL-81	ATCC
BALB/c mice	/	Beijing Vital River Laboratory Animal Technology Co. ,LTD
C57bl/6 mice	/	Jinan Pengyue Experimental Animal Breeding Co. LTD
Rhesus monkeys	/	Hengshu Biotechnology

Supplementary Table 3. Cryo-EM data collection, refinement and validation statistics

	S-CA521^{FALA} IgG Consensus map (EMD-30629)	S-CA521^{FALA} Fab Consensus map (EMD-30951)	S-CA521^{FALA} Fab Focused refinement of Fab-RBD (EMD-30950) (PDB 7E23)
**Data collection and processing**			
Magnification	64k	64k	64k
Voltage (kV)	300	300	300
Electron exposure (e^{-}/Å^2)	48.4	48.4	48.4
Defocus range (μm)	-1.4~-2.4	-1.4~-2.4	-1.4~-2.4
Pixel size (Å)	1.087	1.087	1.087
Symmetry imposed	C1	C1	C1
Initial particle images (no.)	2,796,236	1,487,245	1,487,245
Final particle images (no.)	614,999	328,169	328,169
Map resolution (Å)	3.8	3.0	3.3
FSC threshold	0.143	0.143	0.143
Map resolution range (Å)	3.5-10.0	2.9-10.0	2.6-8.0
**Refinement**			
Initial model used (PDB code)			7CH5
Model resolution (Å)			3.3
FSC threshold			0.143
Model resolution range (Å)			
Map sharpening *B* factor (Å^2)	-150.3	-95.3	-55.9
Model composition			
Non-hydrogen atoms			3133
Protein residues			403
Ligands			1
B factors (Å^2)			
Protein			
Ligand			
R.m.s. deviations			
Bond lengths (Å)			0.006
Bond angles (°)			0.836
Validation			
MolProbity score			2.35
Clashscore			14.03
Poor rotamers (%)			0
Ramachandran plot			

Favored (%)	81.42
Allowed (%)	17.56
Disallowed (%)	1.02

Supplementary Table 4. Starting concentration of analyte (nM) in affinity assay to FcγRs

Starting concentration of analyte (nM)	FcγRI	FcγRIIA R167	FcγRIIA H167	FcγRIIB/C
CA521(IgG1)	40	5000	5000	20000
CA521^{FALA}	40	20000	20000	20000
